# Honoring the legacy of George Sheldrick: Insights from the Cambridge Structural Database

**DOI:** 10.1063/4.0001232

**Published:** 2026-10-27

**Authors:** Suzanna C. Ward, Caroline J. E. Davies-Brooks, Natalie T. Johnson, Matthew P. Lightfoot, Seth B. Wiggin

**Affiliations:** Cambridge Crystallographic Data Centre, 12 Union Road, Cambridge CB2 1EZ, United Kingdom

## Abstract

George M. Sheldrick (1942–2025) transformed the fields of crystallography and structural chemistry. He was a remarkable researcher and educator and developed the SHELX program suite, which will continue to teach and make accessible structural determination of chemical structures at an international level. From the perspective of the Cambridge Structural Database [Groom *et al.*, “The Cambridge Structural Database,” Acta Crystallogr., Sect. B **72**(2), 171–179 (2016)] (CSD), Sheldrick's impact can be seen through his direct contribution of more than 800 crystal structures encompassing a broad range of chemistry, as well as through the widespread adoption of SHELX for crystal structure solution and refinement. For several decades, SHELX has been one of the most widely used software suites used by crystallographers depositing structures in the CSD and has played a central role in the determination of a substantial proportion of the structures contained within the database. Beyond his scientific achievements, Sheldrick was a gifted teacher and mentor whose influence extended across generations of crystallographers worldwide. In this contribution, we reflect on George Sheldrick's career and legacy from the perspective of the CSD, highlighting both his personal contributions to the database and the enduring impact of the SHELX programs on crystallography.

## INTRODUCTION

George M. Sheldrick was one of the most influential crystallographers of the modern era. Throughout a career spanning more than five decades, he made outstanding contributions to structural chemistry as a researcher, software developer, educator, and mentor.^2^ He has over 270 000 citations and an h-index of 116.^3^ His SHELX software suite profoundly shaped small-molecule crystallography as it is today. His 2008 paper, “A Short History of SHELX”^4^ ranks 5th in Nature's top 10 most cited works of the 21st century.^5^

Among his many achievements, the SHELX software suite^6^ has had a profound and enduring impact on crystallography. Its user-friendly design, extensive documentation, and continued support^7^ helped make crystallographic methods accessible to researchers worldwide and Sheldrick has been described as helping to democratize x-ray crystallography.^8^

The Cambridge Structural Database (CSD)^1^ is a trusted resource containing over 1.4 × 10^6^ fully curated organic and metal-organic structures and is maintained by the Cambridge Crystallographic Data Centre (CCDC). The CSD provides a unique perspective from which to examine his legacy. In addition to contributing more than 800 crystal structures to the CSD, Sheldrick's influence can be seen through the widespread use of SHELX by crystallographers depositing structures into the database.

## PERSONAL CONTRIBUTION TO CSD

Sheldrick's career spanned more than five decades, beginning with him studying Natural Sciences at The University of Cambridge, UK in 1960,^9^ incidentally around the time of the inception of the CSD in 1965. Sheldrick directly contributed crystal structures to the CSD and his SHELX software underpins the determination of a significant proportion of entries in the CSD.

Sheldrick coauthored 819 CSD structures determined from a wide range of chemistry research areas. His simplest structure by unit cell volume, trimethylammonium iodide (CSD Refcode TMEAMA,^10^ 323 Å) was published in 1970.^11^ The most complex, a polypeptide structure of the antibiotic mersacidin^12^ (CSD Refcode WOPCAF,^13^ 57 067 Å^3^), was reported in 2000 as the largest approximately equal-atom unknown structure solved by direct methods at that time. This structure is also notable as the merohedrally twinned crystals needed to be “detwinned” prior to structure solution, meaning the determination was a significant achievement. Approximately 57% of his CSD entries are organic in nature, with the remaining containing at least one metal atom; collectively, these structures contain 60 different elements. Figure 1 shows one of his most diverse structures, containing 8 different elements in a single molecule^14^ (CSD Refcode CICLEF^15^).

**FIG. 1.**
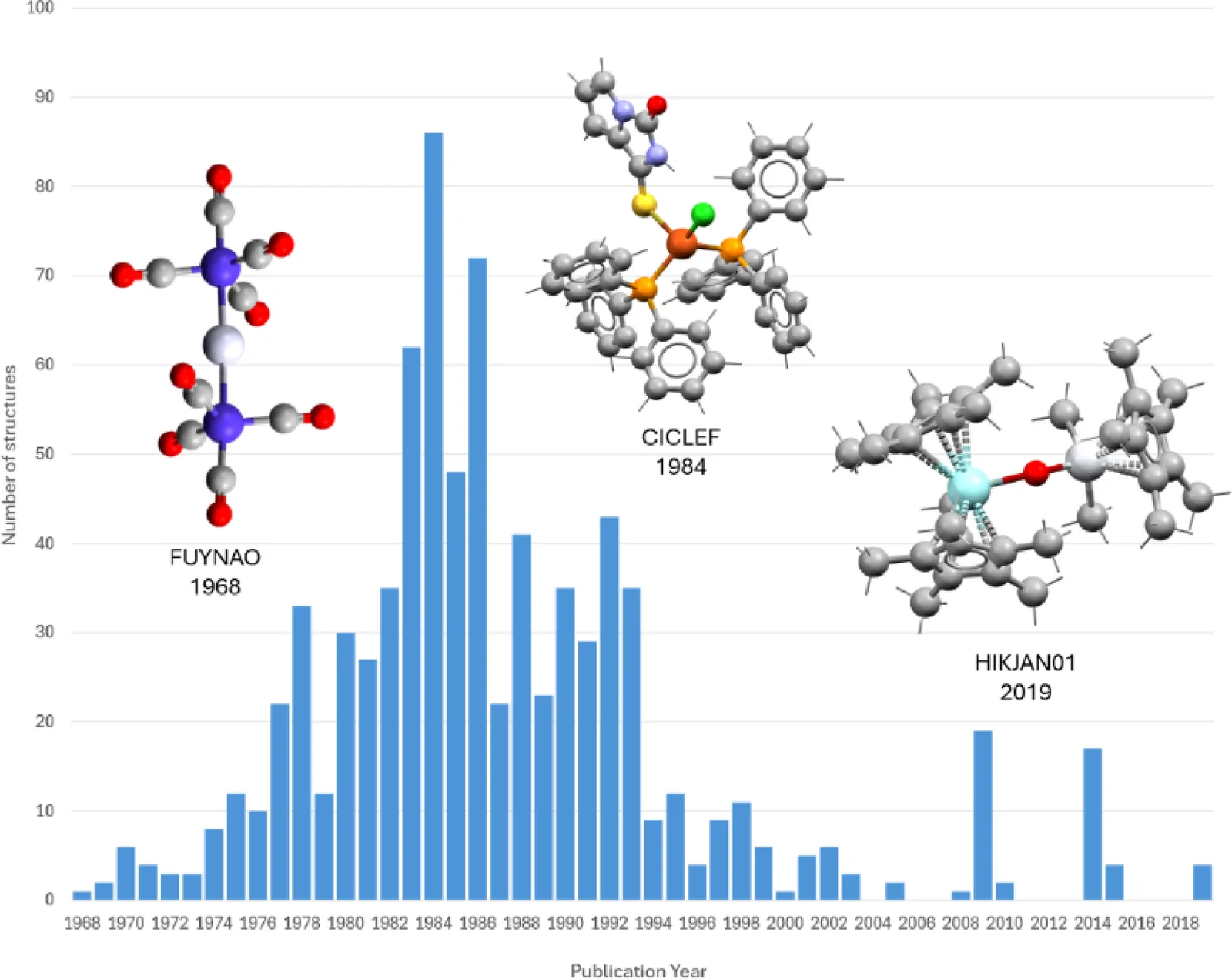
A timeline of George Sheldrick's contributions to the CSD, including images of some notable structures from throughout his career.

The collection includes four neutron diffraction studies and ten determinations from synchrotron diffraction with the remainder from x-ray diffraction. Sheldrick and his coauthors solved most of these structures at room temperature; however, there were several notable exceptions. In particular, one study investigated the temperature dependence of hydrogen atomic displacement parameters H-*U*_iso_ within the riding hydrogen model.^16^ Within this study, 12 structure determinations were reported including Sheldrick's lowest temperature determination, which was an experiment conducted at 9 K (CSD Refcodes POKKAD05^17^ and POKKAD01^18^).

Sheldrick's structures span 68 space groups, and their overall distribution largely reflects that of the CSD. P2_1_/c is the most frequently occurring space group both in the full database (33.8%) and in Sheldrick's subset (32.2%), and the five most common space groups are identical in both cases, albeit in a slightly different order. The average R-factor across his coauthored CSD structures is 5.7%, compared to an average of 5.2% for the CSD. His structure with the lowest reported R-factor of 1.4% was achieved in a single crystal study of a tungsten salt^19^ (CSD Refcode TUWLAZ03^20^) measured using a conventional sealed tube x-ray source.

Sheldrick's first contribution to the CSD was a determination of bis(tetracarbonyl-cobalt)-mercury (CSD Refcode FUYNAO, Fig. 1) published in full in 1968^21^ following a preliminary report in 1967.^22^ This study represented only the second reported structure featuring transition-metal to mercury bonds and was undertaken by Sheldrick and Simpson to elucidate the bonding arrangements of the mercury and cobalt atoms within the structure. Notably, this was published in both the CSD and the Inorganic Crystal Structure Database (ICSD), and in recognition of his contributions to structural mineralogy, the sodium–calcium–fluorocarbonate mineral sheldrickite was named in his honor.^23^ Sheldrick's most recent CSD contributions consist of four refinements of Cp*_2_MeZrOTiMe_2_Cp* (where Cp* is pentamethylcyclopentadienyl) (CSD Refcodes HIKJAN01^24^-04, Fig. 1) published in 2019.^25^ This work provided the successful solution and refinement of a set of non-merohedral twins using what was described at the time as a common strategy. In the same study, Sheldrick and coauthors also reported a mineral structure (ICSD 139864, 139873, 139875, 139877, and 139879) and two protein structures [Protein Data Bank (PDB) 6or0 and 6oqz], all exhibiting non-merohedral twinning; the structure solutions highlighted the broad applicability of Sheldrick's TWINABS program. In total, Sheldrick coauthored 121 entries in the Protein Data Bank^26^ (PDB) and 79 structures in the Inorganic Crystal Structure Database^27^ (ICSD), highlighting his broad expertise across a wide range of chemical disciplines.

Among Sheldrick's most high-profile structures are three published in *Nature*. These include a structure of Vancomycin (CSD Refcode VANCMN^28^) a broad-spectrum antibiotic reported in 1978;^29^ a double helical ribonucleic acid (CSD Refcode IFODUF^30^) reported later that same year; and a synthetic analog of the antibiotic triostin A (CSD Refcode TANDEM20^31^) reported in 1981.^32^ Each of these publications included Olga Kennard, the founder of the CSD, as a coauthor. Sheldrick and Kennard coauthored 22 CSD entries associated with 20 scientific articles between 1973 and 1984, highlighting one of the many influential collaborations established during his early years in Cambridge.

Sheldrick was widely regarded within the crystallographic community for being an ideal collaborator and mentor^8^ and has at least 700 different coauthors in his CSD entries. His most prolific collaboration in terms of CSD structures coauthored was with Herbert W. Roesky. Roesky was a colleague in the Institute of Inorganic Chemistry in Göttingen, Germany, and they maintained a particularly productive and long-standing collaboration. In total, they collaborated on 189 CSD entries (constituting 23% of all Sheldrick's CSD entries) between 1980 and 2014 and were jointly awarded the German Research Foundation's prestigious Gottfried Wilhelm Leibniz Prize^33^ in 1988 for their contributions to inorganic chemistry.

The distribution of coauthorships is characterized by a small number of highly productive collaborators alongside a larger number of less frequent associations. Other prolific collaborators include M. Noltemeyer, P. G. Jones, D. Stalke, and W. Clegg. Over the course of his career, Sheldrick established long-lasting professional and personal connections with many of his collaborators and had at least one scientific collaboration with a close family member. His younger brother, William S. Sheldrick (1945–2015), was a Professor of Analytical Chemistry at Ruhr University in Bochum, Germany, and together they coauthored eight CSD entries reported in seven different publications between 1969 and 1982. The number of coauthors varies over time, with a noticeable increase during the 1980s and 1990s, coinciding with the wider adoption of the SHELX program suite. His collaboration with the greatest number of coauthors, 14 in total, was in 2014 when studying nickel catalysts^34^—which included the structure shown in Fig. 2 (CSD Refcode QETGOO^35^).

**FIG. 2.**
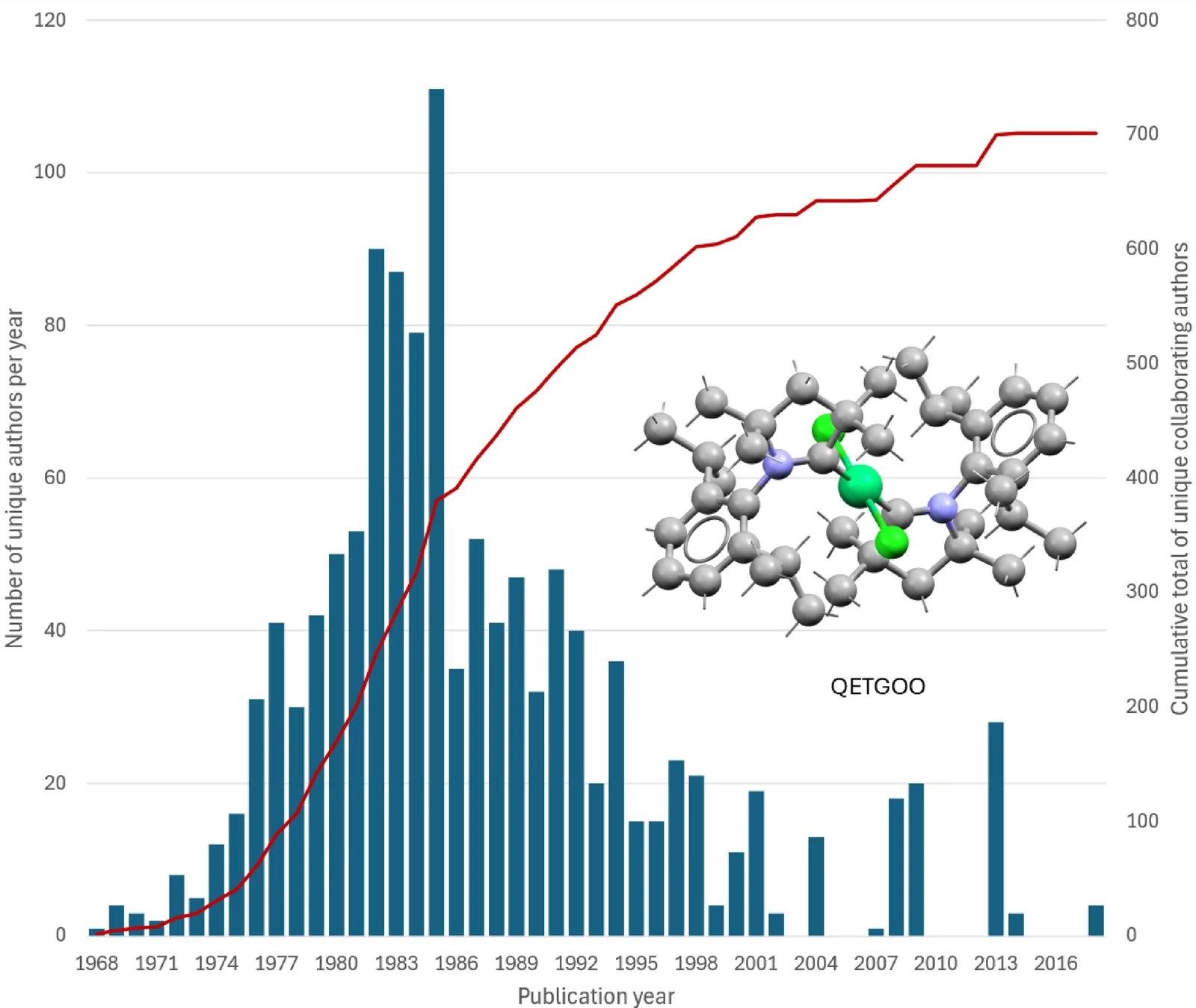
A count of authors who published crystal structures with Sheldrick in the CSD, with the bars showing unique authors per year and the red line showing a cumulative count of collaborating authors.

His collaborations and the journal distribution of CSD entries coauthored by Sheldrick reflects the trajectory of his career, beginning in Cambridge and extending through his long tenure at the University of Göttingen. While this places him firmly within the German and broader European crystallographic communities, the range of journals demonstrates that his collaborations and influence were unmistakably global. Alongside high-impact international titles such as *Nature*, *JACS*, and *Angewandte Chemie*, there is a substantial presence of German-language and German-origin journals, including *Zeitschrift für anorganische und allgemeine Chemie*, *Zeitschrift für Kristallographie*, and *Chemische Berichte*, as well as crystallographic journals established by the International Union of Crystallography (IUCr) such as *Acta Crystallographica*. In total, Sheldrick published CSD structures in over 50 different journals with approximately 12% shared through *Chemische Berichte.*

## CONTRIBUTION TO TEACHING

Sheldrick was widely recognized as an exceptional teacher and mentor whose influence has already extended through several generations of crystallographers. He supervised more than 100 theses,^36^ and many of these individuals continued to publish with him later in their careers, indicating the lasting impact of his role as a mentor.

Within the crystallography community, he was renowned for his ability to explain complex concepts in a clear and compelling manner,^7^ as well as for encouraging critical thinking among his audiences. He demonstrated this not only in his lectures at the institutions where he worked, but also through visiting lectures worldwide, presentations at countless conferences, SHELX workshops, and discussions in both one-to-one and group settings.

His commitment to education and helping others is further exemplified by his personal contribution to the Database for Educational Crystallographic Online Resources (DECOR),^37^ established by Michael Zdilla from Temple University, Pennsylvania, and hosted by the CCDC since 2020. For this collection, Sheldrick shared an extensive set of teaching material that together covers what could be considered a full course in crystallography. The resources shared include slides and practice examples, which span topics ranging from fundamentals such as “*Lattices, Symmetry and Space Groups*” to challenging concepts including anomalous dispersion and modulated structures.

## SHELDRICK'S SOFTWARE CONTRIBUTION TO CSD

The CSD is a vast knowledge base for structural chemistry, and Sheldrick contributed directly to the database with the crystal structures he helped to determine. His legacy also includes the monumental impact of his software, most notably the SHELX software suite, while the widespread use of other programs associated with his work such as XPREP^38^ and SADABS^39^ is readily apparent throughout crystallographic publications.

In his paper “A short history of SHELX,”^4^ Sheldrick comments “After *SHELX* had been used locally in the Cambridge University Chemical Laboratory for several years and so was well tested and debugged, it became clear that it would be useful to have one official definitive final ‘export’ version that would not need any further changes; this version was *SHELX*-76.” Evidence of this can be seen in the CSD; many of Sheldrick's early structures, including the aluminum complex (CSD Refcode MSINAL^40^) published in 1969, feature acknowledgements such as “The calculations were performed using the Titan computer and programs written by G. M. S.,” alluding to this early local usage; but, it is not until 1976 that SHELX is named explicitly in the solution of structures (CSD Refcodes CHOENO^41^ and CYTIDN^42^), with it also referred to as the “SHEL-X program system” (CSD Refcode XACAPU^43^). As SHELX pre-dates the establishment of the CIF syntax^44^ where refinement software usage is recorded in a standardized way, its early usage is challenging to attribute. However, SHELX-76 was soon used by researchers beyond Cambridge, for example in the study of a copper dimer complex (CSD Refcode ICPECU^45^) from researchers from Darmstadt in Germany referencing it in 1978.^44^

Information from the CIF fields of structures deposited at the CCDC allows the contribution of Sheldrick's programs to small-molecule crystallography to be tracked. For the last 20 years, over 90% of CIFs in the CSD contain a Sheldrick reference emphasizing the integral role of his software in crystallographic workflows since its release.

SHELX's comprehensive and clear documentation, regular updates, and availability enabled it to be used by crystallographers worldwide. The features of SHELX's programs helped standardize structural refinement, enabling direct comparison between structures from different authors and research groups.

The widespread usage of SHELX software can also be shown to have been a major contributor to efforts to increase the reliability and reusability of data, with the introduction of structure-factor information. Following a number of retractions of papers due to concerns of the validity of crystal structure data, in 2011 the International Union of Crystallography encouraged researchers to make structure-factor data available.^46^ However, it was the release of SHELXL-2013, where *hkl* and *res* information were automatically embedded in the CIF file, that resulted in a significant increase in this information being included in structures deposited at the CCDC, as shown in Fig. 3. The impact of this continual development of SHELX further emphasizes the important long-term legacy of Sheldrick's software on the scientific community.

**FIG. 3.**
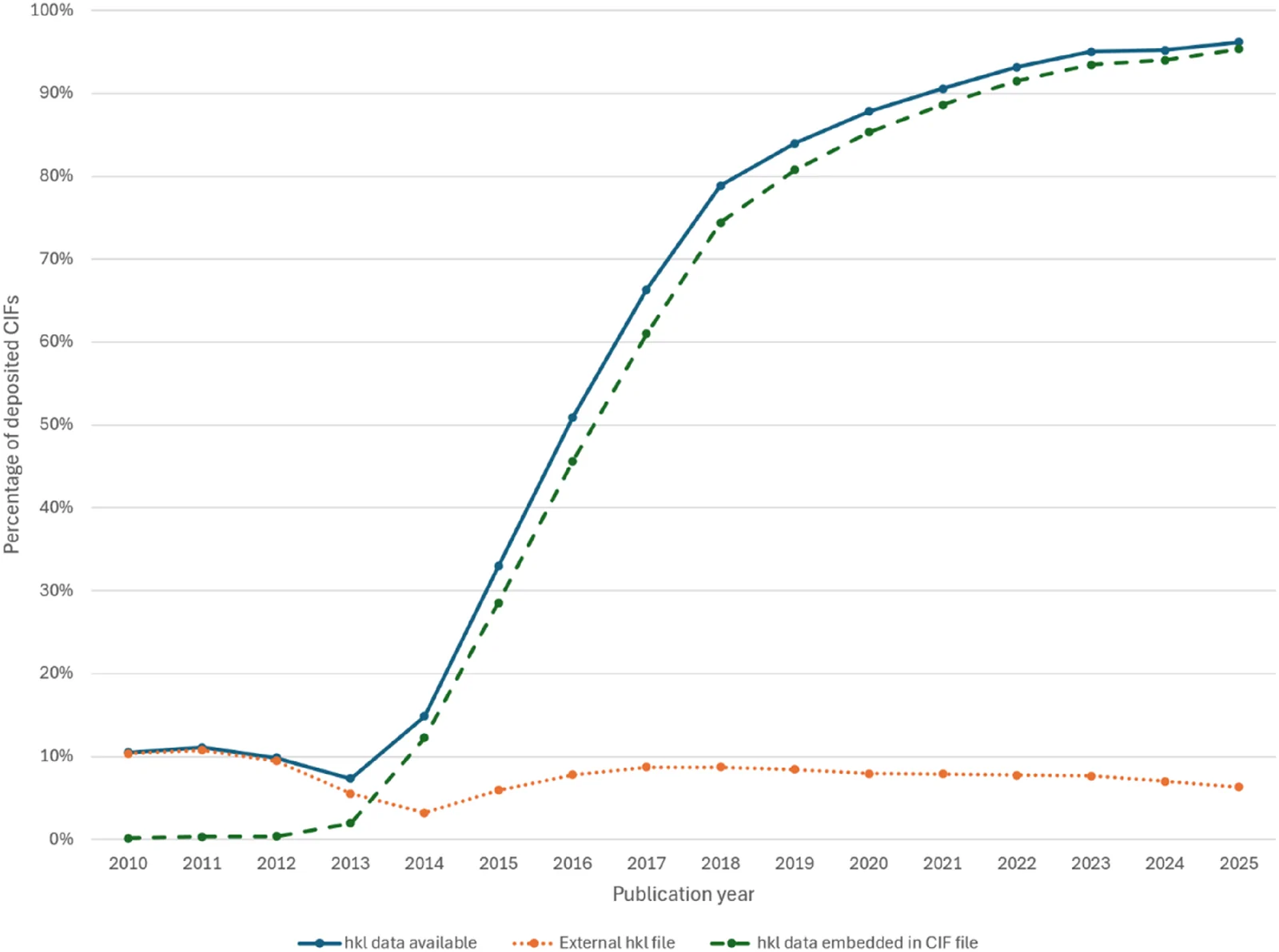
Chart showing the availability of hkl data in deposits to the CSD.

## SUMMARY

Sheldrick's contribution to crystallography was exceptional, both through his own scientific achievements and through the significant influence of the SHELX program suite. Over a career spanning more than five decades, he coauthored more than 800 crystal structures deposited in the CSD, encompassing a wide diversity of chemistry, experimental approaches, and crystallographic challenges. These structures alone represent a substantial and enduring contribution to the structural chemistry community.

The perspective provided by the CSD, however, reveals an even broader impact. Through the widespread adoption of SHELX for crystal structure solution and refinement, Sheldrick's influence extends far beyond the structures that bear his name as author. For many years, SHELX has been the predominant software suite used by crystallographers, underpinning the determination of a substantial percentage of the structures contained within the CSD. The widespread adoption of SHELX throughout the CSD reflects its reliability, accessibility, and ability to evolve alongside advances in experimental crystallography.

Sheldrick's legacy is also evident in the generations of crystallographers he trained and mentored. Through his teaching, workshops, publications, and educational resources, he helped shape crystallographic practices worldwide. Many of the scientists who contribute structures to the CSD today learned crystallography using concepts, methods, and software developed by Sheldrick throughout his career.

The CSD provides a lasting record of Sheldrick's influence on crystallography. His own structures form part of the scientific archive, while SHELX has enabled countless other researchers to contribute high-quality structural data. Few individuals have influenced the contents of the database so profoundly and the continuing use of SHELX ensures that his contributions remain embedded within both the CSD and the wider practice of crystallography.

## Data Availability

Data sharing is not applicable to this article as no new data were created or analyzed in this study.
